# Safety and Efficacy of a Hydrolyzed Collagen Formulation (HYCOS) for Facial Skin Elasticity and Wrinkles: A Randomized, Double‐Blind, Placebo‐Controlled Trial

**DOI:** 10.1111/jocd.71121

**Published:** 2026-08-25

**Authors:** Sutthinee Wisutthathum, Preeyanuch Pimjuk, Nitina Yeesibsean, Katechan Jampachaisri, Panlop Chakkavittumrong, Warisara Pasri, Pichet Kittikun, Supaporn Tuanthai, Thanwarat Suebwongfag, Tanyarat Sutti, Jew Puay Tay, Anjana Fuangchan, Neti Waranuch

**Affiliations:** ^1^ Department of Biochemistry, Faculty of Medical Science Naresuan University Phitsanulok Thailand; ^2^ Cosmetics and Natural Products Research Center (CosNat), Faculty of Pharmaceutical Sciences Naresuan University Phitsanulok Thailand; ^3^ Division of Dermatology, Department of Medicine, Faculty of Medicine Naresuan University Phitsanulok Thailand; ^4^ Department of Mathematics, Faculty of Science Naresuan University Phitsanulok Thailand; ^5^ Division of Dermatology, Department of Medicine, Faculty of Medicine Thammasat University Pathum Thani Thailand; ^6^ Blackmores Institute Warriewood New South Wales Australia; ^7^ Department of Pharmacy Practice, Faculty of Pharmaceutical Sciences Naresuan University Phitsanulok Thailand; ^8^ Department of Pharmaceutical Technology and Center of Excellence for Innovation in Chemistry, Faculty of Pharmaceutical Sciences Naresuan University Phitsanulok Thailand

**Keywords:** hydrolyzed collagen, randomized controlled trial, skin elasticity, skin hydration, skin roughness, wrinkle scale, wrinkles

## Abstract

**Background:**

Oral collagen supplements are widely marketed for improving skin aging parameters, however, high‐quality clinical evidence for multi‐ingredient formulations in Asian populations remains limited.

**Aims:**

This study evaluated the safety and efficacy of a hydrolyzed collagen formulation (HYCOS) on facial skin parameters in healthy Thai volunteers.

**Methods:**

A 12‐week double‐blind, randomized placebo‐controlled trial was conducted in 140 Thai participants aged 30–60 with mild‐to‐moderate facial wrinkles. Participants were received either HYCOS collagen (hydrolyzed collagen with Glutathione and Vitamin E) or a placebo drink. Skin hydration, elasticity, and crow's feet wrinkle roughness were measured using Corneometer, Cutometer, and 3D skin imaging (PRIMOS^CR^), respectively. Dermatologist wrinkle grading and participant self‐assessment were collected. Safety was evaluated through adverse event monitoring and blood/urine biochemical testing.

**Results:**

A total of 135 participants completed the study (test product, *n* = 66; placebo, *n* = 69). After 12 weeks, both groups showed significant increases in skin hydration (*p* < 0.001), with no between‐group difference. The test product group demonstrated greater improvements in skin elasticity; gross elasticity (R2), net elasticity (R5), and overall elasticity (R7) compared with placebo (*p* < 0.05). Crow's feet wrinkle roughness analysis revealed significantly greater reductions in wrinkle roughness in the test product group than in the placebo group at week 12. No product‐related adverse events were observed, and laboratory values remained within normal ranges.

**Conclusion:**

Daily supplementation with HYCOS collagen formula for 12 weeks was safe and well tolerated, with significant improvements in skin elasticity and wrinkle roughness. Increases in skin hydration were observed in both groups, suggesting that this effect may be influenced by hydration‐related or behavioural factors rather than the supplement alone. Overall, HYCOS supplementation may provide beneficial effects on skin aging parameters in healthy adults.

**Trial Registration:**

Thai Clinical Trials Registry: TCTR20250619006

## 1 Introduction

Facial skin aging is driven by a combination of intrinsic processes, including chronological aging and genetic susceptibility, and extrinsic influences such as ultraviolet (UV) radiation, pollution, smoking, and oxidative stress [1, 2, 3]. These factors contribute to structural and functional deterioration of the dermal extracellular matrix (ECM), characterized by fragmentation and reduction of type I collagen, diminished fibroblast activity, increased matrix metalloproteinase (MMP) expression, and progressive loss of elasticity and firmness [2, 4, 5]. Clinically, these molecular changes manifest as fine lines, wrinkles, dryness, and decreased resilience of the skin features that substantially affect appearance, psychosocial well‐being, and quality of life.

Oral hydrolyzed collagen supplementation has emerged as a promising “nutricosmetic” strategy intended to support ECM homeostasis from within [6, 7]. Hydrolyzed collagen peptides, especially those derived from specific bioactive sequences, consist of low‐molecular‐weight peptides that are efficiently absorbed, detectable in plasma, enhances the distribution in tissues [8, 9] and accumulate in the skin where they may stimulate fibroblast proliferation, collagen synthesis, elastin and hyaluronic acid production [10, 11], and inhibit UV‐induced MMP activity [12]. These mechanistic observations support the growing use of collagen peptides as an internal adjunct to conventional topical and procedural anti‐aging interventions. Recent randomized controlled trials (RCTs) have reported improvements in skin hydration, elasticity, and wrinkle appearance following 8–12 weeks of hydrolyzed collagen supplementation compared with placebo [7, 13, 14, 15, 16, 17]. Systematic reviews and meta‐analyzes similarly conclude that oral collagen peptides exert beneficial effects on skin aging parameters, including elasticity and wrinkle depth [18, 19, 20, 21]. However, the magnitude and pattern of benefit can vary depending on peptide source, dose, formulation, and study population. Data in Asian populations, particularly in real‐world nutraceutical products that combine collagen with other ingredients, remain relatively limited. Moreover, few studies have simultaneously evaluated objective biophysical parameters, high‐resolution 3D wrinkle analysis, and patient‐reported outcomes within a single trial.

The test formulation used in the present study contains a combination of hydrolyzed collagen peptides together with glutathione and vitamin E (D‐alpha‐tocopherols succinate). These ingredients are frequently incorporated into commercial nutricosmetic products based on their proposed complementary actions on skin health. Collagen peptides are rich in bioactive collagen‐derived sequences that have been shown to stimulate dermal fibroblast activity and promote extracellular matrix (ECM) synthesis [7, 17]. Glutathione, a key intracellular antioxidant, contributes to redox homeostasis and melanogenesis regulation [22, 23], whereas vitamin E is a potent lipophilic antioxidant that protects cellular membranes from oxidative injury [24]. Despite the growing popularity of such multi‐ingredient formulations, the combined effects of collagen peptides with glutathione and vitamin E on clinically relevant facial skin aging outcomes remain insufficiently characterized. There are few published randomized controlled trials (RCTs) that have evaluated these ingredients together using robust, standardized dermatological endpoints.

This study aimed to evaluate the safety and efficacy of a commercially formulated hydrolyzed collagen product (HYCOS) in healthy Thai volunteers for 12 weeks. A 12‐week, randomized, double‐blind, placebo‐controlled trial was conducted to determine whether daily HYCOS intake improves facial skin hydration, elasticity, and wrinkle parameters compared with a matched placebo drink. Participant satisfaction and safety were assessed through clinical monitoring and laboratory evaluations. We hypothesized that HYCOS would produce greater improvements in skin hydration, elasticity, and wrinkle outcomes relative to placebo, without compromising safety. By integrating a standardized collagen peptide formulation with antioxidant components and applying rigorous clinical methodology consistent with contemporary trial standards, this study aims to provide high‐quality evidence supporting the use of nutricosmetics for managing facial skin aging.

## 2 Methods

### 2.1 Clinical Study Design and Ethics

This study was conceptualized as a single‐center, randomized, double‐blind, placebo‐controlled clinical trial conducted over 12 weeks. The study complied with the Declaration of Helsinki and Good Clinical Practice (ICH‐GCP). The protocol was approved by the Naresuan University Institutional Review Board (IRB) on 30 June 2022 (Certificate of Approval No. 0246/2022; IRB No. P1‐0070/2565), and the trial was conducted between July 2022 to July 2025. All participants were provided with written informed consent prior to any study procedures. The trial was registered in the Thai Clinical Trials Registry: TCTR20250619006, (https://www.thaiclinicaltrials.org/show/TCTR20250619006), a member of the WHO Primary Network of ICTRP. The trial protocol, statistical analysis plan, source documents, and study records were securely maintained at the Cosmetics and Natural Products Research Center (CosNat), Faculty of Pharmaceutical Sciences, Naresuan University.

### 2.2 Recruitment and Eligibility Criteria

Participants were recruited through public advertisements and subsequently screened at the Cosmetics and Natural Products Research Center (CosNat), Faculty of Pharmaceutical Sciences, Naresuan University, Phitsanulok, Thailand. Screening procedures included completion of questionnaires, physical examination, facial wrinkle assessment performed by trained dermatologists, and laboratory investigations. Eligibility was determined according to the following criteria.

#### 2.2.1 Inclusion Criteria

**FIGURE 1:**
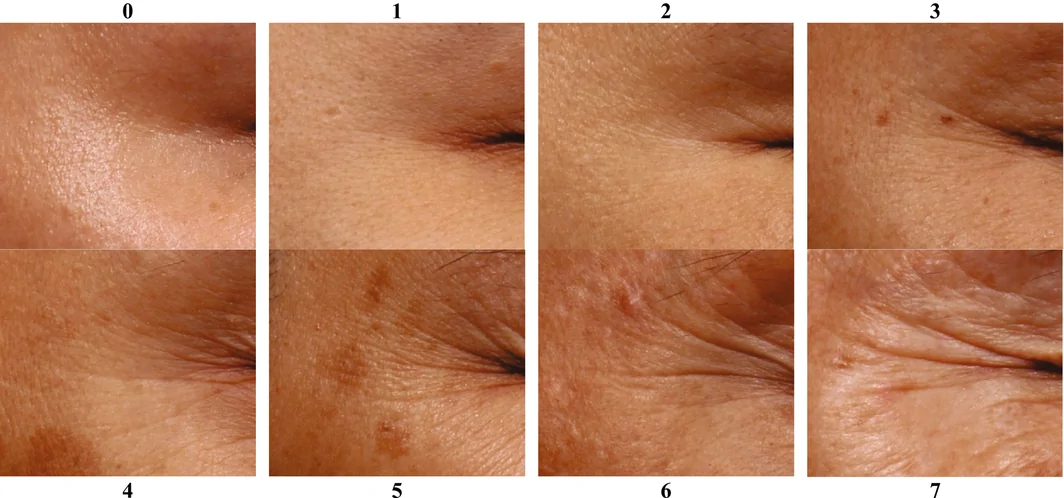
Reference photographs of the CosNat Wrinkle Scale (grades 0–7) used for clinical grading of wrinkle severity. Wrinkle grades are assigned according to the number, depth, and overall morphological appearance of visible wrinkle lines.

Eligible participants were healthy Thai adults aged 30–60 years with any skin type (normal, dry, oily, or combination) who presented with crow's feet wrinkles graded 2–7 according to the standardized wrinkle grading scale developed by the research center (Figure 1).

#### 2.2.2 Exclusion Criteria

Participants were excluded if they had dermatologic conditions at the test sites (e.g., active eczema, psoriasis, scars, or sunburn), significant gastrointestinal or severe indigestion, immunological, or systemic diseases (including cancer, acute cardiac disorders, uncontrolled diabetes), severe skin diseases within the past 6 months, major surgery within the past year, recent use of anti‐inflammatory drugs, antibiotics, or antihistamines (within 4 weeks), known hypersensitivity to product ingredients, BMI > 27 kg/m^2^, pregnancy or lactation, or recent excessive UV exposure at the test sites.

#### 2.2.3 Withdrawal Criteria

Participants were withdrawn in cases of product‐related allergic reactions or serious adverse events, pregnancy during the study, non‐compliance (< 80% adherence or ≥ 5 consecutive missed doses), excessive UV exposure, loss to follow‐up, or voluntary withdrawal.

### 2.3 Interventions

The test product (HYCOS hydrolyzed collagen formula) used in this study contained a combination of hydrolyzed collagen peptides (5000 mg), glutathione (250 mg), and vitamin E (D‐alpha‐tocopherol succinate; 11.5 mg), along with other excipients (citric acid anhydrous, orange flavor, maltodextrin, and stevia leaf extract), totalling 6.5 g per sachet. The placebo product contained only the excipients (maltodextrin, citric acid anhydrous, orange flavor, and stevia leaf extract), also totalling 6.5 g per sachet, and was formulated in indistinguishable visually and organoleptically from the active product. Both the active and placebo products were supplied by the Blackmores Institute, Australia. Participants were instructed to dissolve one sachet in 150–200 mL of room‐temperature or cold water and consumed once daily at any convenient time, for 12 consecutive weeks. They were advised not to initiate new oral supplements or topical anti‐aging treatments, and to avoid excessive sun exposure or tanning procedures during the study. Study products were dispensed at each scheduled study visit (baseline, week 4, and week 8) and participants received a sufficient supply of sachets to last until the next evaluation visit. Participants were instructed to record daily product intake in a study diary and to return all used and unused sachets at each follow‐up visit. Compliance was assessed by reviewing the diary records and counting returned sachets. Compliance (%) was calculated as: (Number of sachets consumed/Number of sachets expected to be consumed) × 100. Participants were considered non‐compliant if their adherence rate was below 80% or if they missed five or more consecutive doses, and were withdrawn according to the predefined study criteria.

### 2.4 Randomization and Blinding

Eligible participants were equally randomized in a parallel‐group design to receive either HYCOS collagen or placebo using a computer‐generated random sequence prepared by an independent statistician. Investigational products were provided in identical single‐dose sachets, labeled as Product A or Product B. Participants, investigators, dermatologists, and study staff involved in data collection and outcome assessment were all blinded to treatment allocation throughout the study. The randomization code was kept by the assigned staff who was not involved in measurement and data analysis process and was only broken after database lock. Each participant was assigned a unique alphanumeric study code, and all data were de‐identified to ensure confidentiality.

### 2.5 Outcomes

All outcome assessments were performed at baseline (week 0) and at 4, 8, and 12 weeks of supplementation. All participants were measured by biophysical instruments in a controlled environment (25°C ± 2°C, relative humidity 55% ± 5%).

#### 2.5.1 Skin Hydration

Facial skin hydration was measured on the left cheek using a Corneometer CM 825 (Courage‐Khazaka, Germany) which determines the stratum corneum moisture content by capacitance. Three readings were taken at each visit and averaged. Higher values indicate higher stratum corneum moisture.

#### 2.5.2 Skin Elasticity

Skin elasticity was assessed using a Cutometer MPA 580 (Courage‐Khazaka, Germany). Measurements were performed on the right cheeks under controlled environmental conditions. Three consecutive suction‐relaxation cycles were applied using a negative pressure of 450 mbar, with 2 s of suction followed by 2 s of relaxation per cycle. Three measurements were obtained at each assessment site and averaged for analysis. From the deformation curves, the following parameters were calculated: Gross elasticity (R2), reflecting the overall ability of the skin to return to its original position after deformation (overall elastic recovery); Net elasticity (R5), representing pure elastic return; Viscoelastic ratio (R6); reflecting quantifies how much of the deformation is due to viscous (slow, plastic‐like) rather than elastic behavior; Portion of the elasticity compared to the complete curve (R7), the proportion of total deformation that is recoverable. Higher values of R2, R5, and R7 indicate greater skin elasticity. In contrast, smaller R6 values reflect improved viscoelastic recovery and therefore correspond to higher overall elasticity.

#### 2.5.3 Skin Wrinkle and Roughness Assessment

Crow's feet wrinkles were measured using a three‐dimensional optical profilometry system, the PRIMOS^CR^ device, equipped with PRIMOS software version 5.8 (GFMesstechnik GmbH, Teltow, Germany). Standardized images of the periocular region were captured, and software‐derived roughness parameters were calculated, including arithmetic roughness average (Ra); reflecting overall surface irregularity or skin roughness; maximal roughness (Rmax); representing the deepest wrinkle feature, total roughness (Rt); indicating the cumulative vertical variation within the skin profile, and an initial roughness (R3z); capturing prominent irregularities in the uppermost skin segments. A decrease in all parameters indicates an improvement in skin roughness and an anti‐wrinkle effect. Additionally, standardized high‐resolution photographs of the crow's feet area were taken under consistent lighting. Blinded evaluators then performed clinical grading of wrinkle severity from these photographs using the CosNat Wrinkle Scale (Grades 0–7). Each photograph was visually compared with the standardized reference photographs before assigning the corresponding wrinkle grade. The grading criteria were defined as follows, based on the number, depth, and overall morphological appearance of visible wrinkle lines: 0 = No wrinkles (Smooth skin surface with uniform micro‐relief and no visible wrinkle lines); 1 = Few slight wrinkles (Barely perceptible superficial fine lines); 2 = Slight wrinkles (One or two distinct superficial fine lines); 3 = Mild wrinkles (One or two distinct superficial wrinkles without depth wrinkle formation); 4 = Mild‐to‐moderate wrinkles (Superficial wrinkles with distinct deep wrinkle formation); 5 = Moderate wrinkles (Multiple deep wrinkles with wrinkle network); 6 = Marked wrinkles (Prominent deep wrinkles with pronounced skin folding), 7 = Severe wrinkles (Prominent deep wrinkles with extensive skin folding and laxity). Representative photographs for each grade are shown in Figure 1. Five trained, independent, blinded evaluators scored each image, and the final clinical wrinkle grade was determined by majority agreement among the five evaluators. These visual assessments were performed at each study visit (baseline and weeks 4, 8, and 12) to complement the objective instrumental measurements.

#### 2.5.4 Participant Self‐Assessment

At week 4, 8 and 12, participants completed a structured self‐assessment questionnaire consisting of 14 items evaluating perceived changes in skin appearance and product acceptability. The questionnaire assessed participants' perceptions regarding skin youthfulness (Q1), hydration (Q2), firmness (Q3), elasticity (Q4), wrinkle appearance (Q5), smoothness (Q6), radiance (Q7), overall skin condition (Q8), taste (Q9), color (Q10), ease of consumption (Q11), gastrointestinal tolerability (Q12), absence of adverse effects (Q13), and overall product satisfaction (Q14). Responses were recorded using a 5‐point Likert scale ranging from 1 (strongly disagree) to 5 (strongly agree).

#### 2.5.5 Safety Assessments

Safety evaluations included vital signs and physical examinations at each study visit. Laboratory tests (complete blood count, liver, and renal function tests, fasting blood glucose, and urinalysis) were performed at baseline and week 12. Participants maintained diaries to record any symptoms or adverse events. The study physicians reviewed diaries and interviewed subjects at each visit to inquire about potential adverse events (e.g., gastrointestinal issues, allergic reactions, skin irritation). Product adherence was assessed by counting unused sachets returned at each visit.

### 2.6 Sample Size Calculation

The sample size was calculated using 2 groups with 4 repeated measurements via G Power program, with the effect size of 0.01, 0.05 significance level. We obtained the total sample size of 114 (57 in each group) with the 80% power of analysis. To compensate for 22% dropout rate, the required sample size is 140 (70 in each group).

### 2.7 Statistical Analysis

Statistical analyzes were performed using SPSS version 23. Descriptive statistics, including frequencies, percentages, means, and standard deviations, were used to summarize participant characteristics and outcome variables. The Shapiro–Wilk test was applied to assess the normality of continuous data. Depending on distributional assumptions and variable type, within‐group comparisons were conducted using paired *t*‐tests, while between‐group differences were evaluated using independent *t*‐tests, one‐way ANOVA, or analysis of covariance when adjustment for baseline values was required. Associations between categorical variables were analysed using Chi‐square or Fisher's exact tests, as appropriate. A one‐side or two‐sided *p*‐value of < 0.05 was considered statistically significant. Efficacy analyzes were performed on participants who completed the study with complete outcome data (per‐protocol population). Participants who withdrew, were lost to follow‐up, or had incomplete data were excluded from the final efficacy analysis, and no imputation of missing data was performed. Safety analyzes were conducted on an intention‐to‐treat basis, including all participants who received at least one dose of the study product.

## 3 Results

### 3.1 Participant Flow and Baseline Characteristics

**FIGURE 2:**
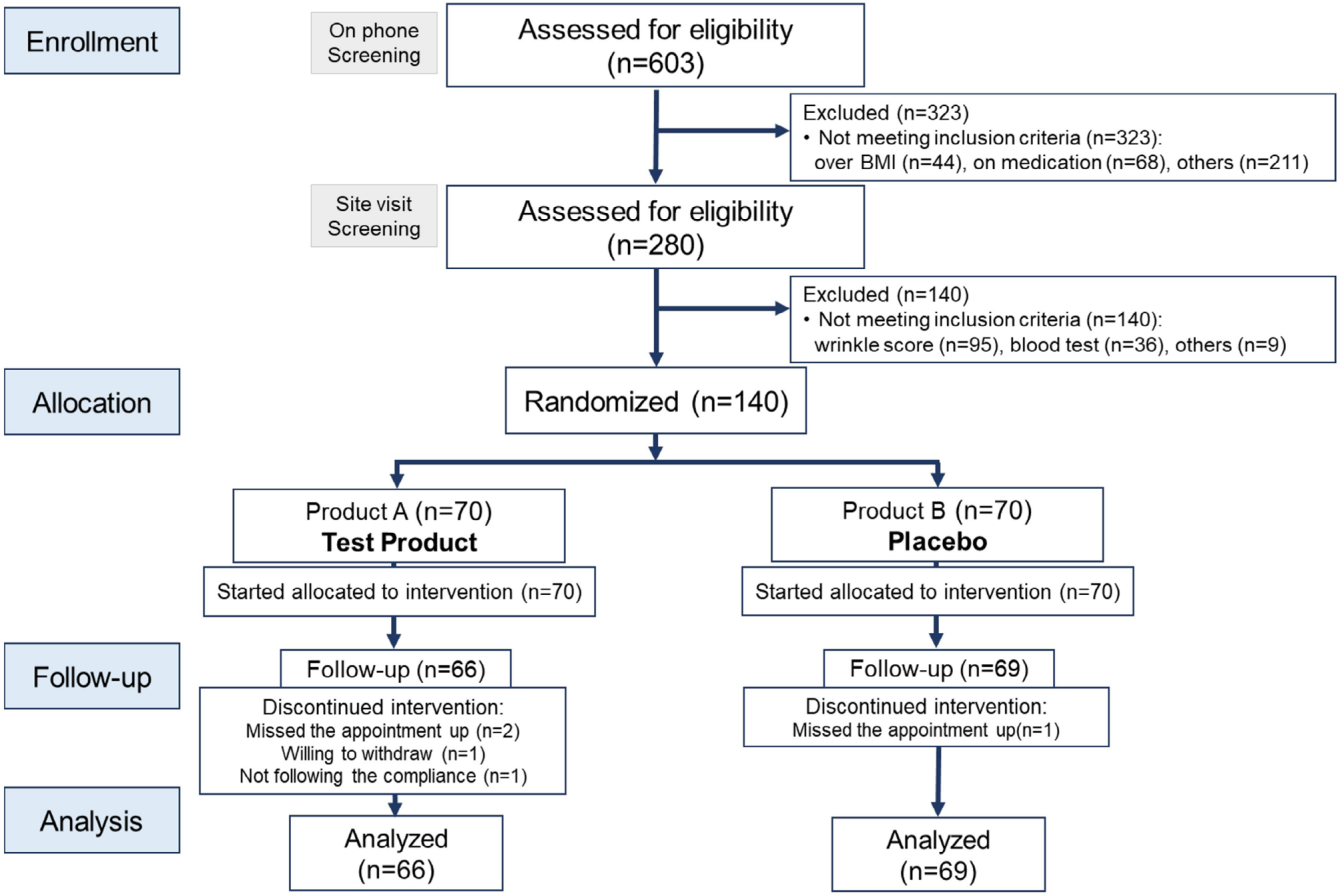
CONSORT flowchart of participant enrolment in the study.

A total of 140 participants were enrolled and randomized (70 to test product, 70 to placebo) as showed in Figure 2. Five participants did not complete the study (four in the test product group and one in the placebo group) due to loss to follow‐up or non‐compliance. Therefore, 135 participants (66 test product, 69 placebo) completed the 12‐week intervention and were included in the analysis.

Baseline demographic and clinical characteristics were similar between groups (Table 1). The mean age was approximately 45 years in both groups, with roughly three‐quarters female. Mean body mass index was in the normal‐to‐slightly‐overweight range. Facial skin type distribution and baseline wrinkle severity scores were comparable. Baseline Corneometer hydration values and Cutometer elasticity parameters did not differ significantly between treatment arms.

**TABLE 1:** Demographics and baseline characteristics of the participants.

Characteristics	Test product (*n* = 66)	Placebo (*n* = 69)	*p*
	*n* (%)	*n* (%)	
Age (Mean ± SD)	45.65 ± 6.37	45.10 ± 6.38	0.62^a^
BMI (Mean ± SD)	23.49 ± 2.34	23.68 ± 2.73	0.67^a^
Gender			0.65^b^
Female	52 (78.8)	52 (75.4)	
Male	14 (21.2)	17 (24.6)	
Facial skin types			0.74^b^
Normal	9 (13.6)	10 (14.5)	
Dry	17 (25.8)	21 (30.4)	
Oily	10 (15.2)	13 (18.8)	
Combination	30 (45.5)	25 (36.2)	
Facial skin sagging			0.61^b^
Slight	36 (54.5)	41 (59.4)	
Moderate	26 (39.4)	22 (31.9)	
Extreme	4 (6.1)	6 (8.7)	
Depth of wrinkles around the corners of the eyes			0.29^b^
Level 2	29 (43.9)	40 (58.0)	
Level 3	25 (37.9)	15 (21.7)	
Level 4	8 (12.1)	8 (11.6)	
Level 5	4 (6.1)	4 (5.8)	
Level 6	0	1 (1.4)	
Level 7	0	1 (1.4)	

Skin parameters	Mean ± SD	Mean ± SD	*p* ^b^
Skin moisture	60.37 ± 13.23	57.64 ± 13.88	0.138^a^
Skin elasticity			
Gross elasticity (R2)	0.537 ± 0.056	0.564 ± 0.065	0.994^a^
Net elasticity (R5)	0.480 ± 0.067	0.485 ± 0.066	0.667^a^
Viscoelastic ratio (R6)	0.694 ± 0.089	0.677 ± 0.126	0.816^a^
Portion of elasticity (R7)	0.283 ± 0.040	0.289 ± 0.036	0.832^a^
Skin roughness and wrinkles			
Arithmetic roughness average (Ra)	25.94 ± 5.42	26.29 ± 5.11	0.352^a^
Maximal roughness (Rmax)	202.97 ± 46.63	205.54 ± 41.61	0.368^a^
Total roughness (Rt)	214.87 ± 49.60	217.73 ± 44.23	0.362^a^
Initial roughness (R3z)	71.62 ± 18.31	75.74 ± 16.20	0.084^a^

^a^ Independent *t*‐test.

^b^ Chi‐square test.

### 3.2 Skin Hydration

Facial skin hydration increased significantly from baseline in both groups at week 12 (Table 2). In the test product group, mean hydration values range from 60.37 ± 13.23 at baseline to 65.11 ± 12.06 at week 12 (*p* < 0.05 vs. baseline). Similar increases were observed in the placebo group from 57.64 ± 13.88 at baseline to 63.09 ± 13.73 at week 12 (*p* < 0.05 vs. baseline). However, no statistically significant differences between groups were detected at any time point.

**TABLE 2:** Comparison skin moisture content of the test product and placebo.

Skin moisture	Test product (*n* = 66)	Placebo (*n* = 69)	*p* ^a^
	Mean ± SD	Mean ± SD	
Baseline	60.37 ± 13.23	57.64 ± 13.88	0.138
Week 4	60.38 ± 13.18	58.65 ± 13.66	0.311
*p* ^b^ (Baseline vs. Week 4)	0.647	0.163	
Week 8	61.80 ± 11.63	58.24 ± 13.86	0.098
*p* ^b^ (Baseline vs. Week 8)	0.205	0.301	
Week 12	65.11 ± 12.06^#^	63.09 ± 13.73^#^	0.194
*p* ^b^ (Baseline vs. Week 12)	< 0.001	< 0.001	

^a^ One‐tailed test denotes the test for (mean of Test Product − mean of Placebo) is greater than zero using independent *t*‐test.

^b^ One‐tailed test denotes the test for (mean of other weeks − mean of Week 0) is greater than zero using a paired *t*‐test, where ^#^ denote statistically significant with *p*‐value < 0.05 for one‐tailed test.

### 3.3 Skin Elasticity

Skin elasticity parameters, including gross elasticity (R2), net elasticity (R5), viscoelastic ratio (R6), and portion of the elasticity compared to the complete curve (R7), were evaluated over the 12‐week intervention (Table 3). At baseline, values were comparable between the test product and placebo groups across all parameters. *Gross elasticity (R2)*: In the test product group, R2 increased significantly from baseline at week 4 and week 8 (*p* < 0.05), with values rising from 0.537 ± 0.056 at baseline to 0.560 ± 0.069 and 0.556 ± 0.070, respectively. By week 12, the increase remained higher than baseline, although the change did not reach statistical significance. The placebo group showed minimal change over time, with no significant within‐group improvements noted. Between‐group comparisons showed that R2 was significantly higher in the test product group at week 4 (*p* < 0.05), indicating an earlier improvement in elasticity relative to placebo. *Net elasticity (R5)*: participants receiving the test product exhibited a significant improvement in R5 at week 4 (0.506 ± 0.079 vs. 0.480 ± 0.067 at baseline; *p* < 0.05). R5 values continued to show a positive upward trend through weeks 8 and 12, despite the changes not attaining statistical significance. In contrast, the placebo group showed no significant within‐group improvements in R5 during weeks 4 and 8, with values exhibiting a slight downward trend. At week 12, a statistically significant increase from baseline was observed (0.503 ± 0.068 vs. 0.485 ± 0.066 at baseline; *p* < 0.05). Between‐group comparison revealed a higher R5 value in the test product group at week 4 (*p* < 0.05). *Viscoelastic Ratio (R6)*: R6 values insignificantly change across all time points in both groups. *Portion of the elasticity compared to the complete curve (R7)*: the test product group demonstrated a significant increase in R7 values at week 4, rising from 0.283 ± 0.040 at baseline to 0.294 ± 0.043 (*p* < 0.05). R7 values continued to exhibit a gradual increasing trend at week 8 and 12, although these incremental improvements did not reach statistical significance. The placebo group did not exhibit notable changes in R7 across the study period. Between‐group comparison at week 4 showed a tendency toward higher R7 values in the test product group (*p* = 0.067), although the difference did not reach statistical significance.

**TABLE 3:** Comparison skin elasticity of the test product and placebo.

Skin elasticity	Test product (*n* = 66)	Placebo (*n* = 69)	*p* ^a^
	Mean ± SD	Mean ± SD	
Gross elasticity (R2)			
Baseline	0.537 ± 0.056	0.564 ± 0.065	0.994
Week 4	0.560 ± 0.069*^#^	0.541 ± 0.062	0.046
*p* ^b^ (Baseline vs. Week 4)	0.004	0.999	
Week 8	0.556 ± 0.070^#^	0.547 ± 0.060	0.221
*p* ^b^ (Baseline vs. Week 8)	0.028	0.975	
Week 12	0.554 ± 0.068	0.566 ± 0.060	0.861
*p* ^b^ (Baseline vs. Week 12)	0.081	0.385	
Net elasticity (R5)			
Baseline	0.480 ± 0.067	0.485 ± 0.066	0.667
Week 4	0.506 ± 0.079*^#^	0.483 ± 0.066	0.034
*p* ^b^ (Baseline vs. Week 4)	0.003	0.628	
Week 8	0.488 ± 0.078	0.479 ± 0.072	0.238
*p* ^b^ (Baseline vs. Week 8)	0.174	0.751	
Week 12	0.493 ± 0.079	0.503 ± 0.068^#^	0.792
*p* ^b^ (Baseline vs. Week 12)	0.099	0.021	
Viscoelastic ratio (R6)			
Baseline	0.694 ± 0.089	0.677 ± 0.126	0.816
Week 4	0.718 ± 0.104	0.698 ± 0.114	0.852
*p* ^b^ (Baseline vs. Week 4)	0.955	0.943	
Week 8	0.706 ± 0.086	0.693 ± 0.104	0.780
*p* ^b^ (Baseline vs. Week 8)	0.856	0.892	
Week 12	0.698 ± 0.095	0.710 ± 0.111	0.261
*p* ^b^ (Baseline vs. Week 12)	0.602	0.984	
Portion of elasticity (R7)			
Baseline	0.283 ± 0.040	0.289 ± 0.036	0.832
Week 4	0.294 ± 0.043^#^	0.284 ± 0.033	0.067
*p* ^b^ (Baseline vs. Week 4)	0.010	0.936	
Week 8	0.286 ± 0.045	0.283 ± 0.037	0.940
*p* ^b^ (Baseline vs. Week 8)	0.270	0.940	
Week 12	0.291 ± 0.041	0.295 ± 0.036	0.130
*p* ^b^ (Baseline vs. Week 12)	0.083	0.130	

^a^ One‐tailed test denotes the test for (mean of Test Product − mean of Placebo) is greater than zero for R2, R5, R7 or smaller than zero for R6 using independent *t*‐test, where * denote statistically significant with *p*‐value < 0.05 for one‐tailed test.

^b^ One‐tailed test denotes the test for (mean of other weeks − mean of Week 0) is greater than zero for R2, R5, R7 or smaller than zero for R6 using a paired *t*‐test, where ^#^ denote statistically significant with *p*‐value < 0.05 for one‐tailed test.

### 3.4 Skin Roughness and Wrinkles

Skin roughness and wrinkle parameters, including arithmetic roughness (Ra), maximum roughness (Rmax), total roughness (Rt), and initial roughness (R3z), were assessed over 12 weeks (Table 4). Baseline values were comparable between groups for all parameters, indicating similar initial wrinkle severity. *Arithmetic Roughness (Ra)*: In the test product group, Ra values significantly decreased to 24.80 ± 5.84, 24.73 ± 5.60 μm, and 24.48 ± 5.30 μm at weeks 4, 8, and 12, respectively, compared with baseline (*p* < 0.05 for all timepoints). Similarly, in the placebo group, Ra values also significantly decreased to 25.62 ± 4.98 μm, 25.01 ± 4.48 μm, and 25.29 ± 4.58 μm at weeks 4, 8, and 12, respectively, compared with baseline (*p* < 0.05 for all timepoints). Improvements in Ra were observed in both the test product and placebo groups across all time points, although between‐group comparisons did not reveal any significant differences during the study. *Maximum roughness (Rmax)*: Rmax decreased over time in the test product group, with a trend toward improvement at week 4 (202.97 ± 46.63 vs. 196.75 ± 54.26 at baseline; *p* = 0.066), followed by significant reductions at week 8 (192.56 ± 49.88; *p* < 0.001) and week 12 (193.93 ± 47.09; *p* < 0.05). These changes indicate peak reduction of wrinkle depth. In the placebo group, Rmax decreased from 205.54 ± 41.61 (at baseline) to 200.86 ± 39.77, 197.16 ± 36.24, and 200.30 ± 38.71 at weeks 4, 8, and 12, respectively. The significant reduction was observed in comparing at week 8 with baseline (*p* < 0.05). No between‐group differences reached statistical significance. *Total roughness (Rt)*: Rt progressively declined in the test product group throughout the 12‐week period. At week 4, Rt showed a modest reduction from baseline (214.87 ± 49.60 to 208.15 ± 57.47; *p* = 0.064). More pronounced decreases were observed at week 8 (203.99 ± 52.33; *p* < 0.001) and week 12 (205.31 ± 49.19; *p* < 0.05), reflecting an overall improvement in the depth variation of the skin surface. In the placebo group, Rt values also declined over time, decreasing from 217.73 ± 44.23 at baseline to 212.56 ± 41.96, 208.89 ± 38.26, and 211.89 ± 41.06 at weeks 4, 8, and 12, respectively. A statistically significant change was detected only at week 8 (*p* < 0.05). No between‐group differences reached statistical significance. *Initial roughness (R3z)*: a progressive decrease was seen in the test product group over the 12‐week study period. R3z values decreased from 71.62 ± 18.31 at baseline to 69.01 ± 19.70 (*p* < 0.05), 69.33 ± 19.08 (*p* < 0.05), and 67.98 ± 18.39 (*p* < 0.001) at weeks 4, 8, and 12, respectively. In the placebo group, R3z values decreased from 75.74 ± 16.20 at baseline to 74.85 ± 15.57, 73.05 ± 14.46, and 74.38 ± 14.07 at weeks 4, 8, and 12, respectively. A statistically significant change was detected only at week 8 (*p* < 0.05). Between‐group comparisons showed that reductions in R3z values were significantly greater in the test product group than in the placebo group at both week 8 (69.01 ± 19.70 test product vs. 74.85 ± 15.57 placebo; *p* < 0.05) and week 12 (67.98 ± 18.39 test product vs. 74.38 ± 14.07 placebo; *p* < 0.05). *Wrinkle grading*: wrinkle severity was also evaluated using a 7‐point CosNat wrinkle grading scale (Table 5), which showed only minimal changes over the study period. Mean wrinkle scores decreased slightly from baseline to week 12 in both the test product group (2.76 ± 0.88 to 2.67 ± 0.91; *p* = 0.03) and the placebo group (2.68 ± 0.87 to 2.62 ± 0.84; *p* = 0.03). No significant differences were observed between groups at weeks 4, 8, or 12 (*p* > 0.60 for all comparisons).

**TABLE 4:** Comparison skin roughness and wrinkles of the test product and placebo.

Skin roughness and wrinkles	Test product (*n* = 66)	Placebo (*n* = 69)	*p* ^a^
	Mean ± SD	Mean ± SD	
Arithmetic roughness average (Ra)			
Baseline	25.94 ± 5.42	26.29 ± 5.11	0.352
Week 4	24.80 ± 5.84^#^	25.62 ± 4.98^#^	0.191
*p* ^b^ (Baseline vs. Week 4)	0.002	0.021	
Week 8	24.73 ± 5.60^#^	25.01 ± 4.48^#^	0.378
*p* ^b^ (Baseline vs. Week 8)	< 0.001	< 0.001	
Week 12	24.48 ± 5.30^#^	25.29 ± 4.58^#^	0.172
*p* ^b^ (Baseline vs. Week 12)	< 0.001	< 0.001	
Maximal roughness (Rmax)			
Baseline	202.97 ± 46.63	205.54 ± 41.61	0.368
Week 4	196.75 ± 54.26	200.86 ± 39.77	0.308
*p* ^b^ (Baseline vs. Week 4)	0.066	0.084	
Week 8	192.56 ± 49.88^#^	197.16 ± 36.24^#^	0.272
*p* ^b^ (Baseline vs. Week 8)	< 0.001	0.005	
Week 12	193.93 ± 47.09^#^	200.30 ± 38.71	0.196
*p* ^b^ (Baseline vs. Week 12)	0.006	0.073	
Total roughness (Rt)			
Baseline	214.87 ± 49.60	217.73 ± 44.23	0.362
Week 4	208.15 ± 57.47	212.56 ± 41.96	0.305
*p* ^b^ (Baseline vs. Week 4)	0.064	0.083	
Week 8	203.99 ± 52.33^#^	208.89 ± 38.26^#^	0.269
*p* ^b^ (Baseline vs. Week 8)	< 0.001	0.005	
Week 12	205.31 ± 49.19^#^	211.89 ± 41.06	0.200
*p* ^b^ (Baseline vs. Week 12)	0.006	0.065	
Initial roughness (R3z)			
Baseline	71.62 ± 18.31	75.74 ± 16.20	0.084
Week 4	69.01 ± 19.70*^#^	74.85 ± 15.57	0.029
*p* ^b^ (Baseline vs. Week 4)	0.014	0.165	
Week 8	69.33 ± 19.08^#^	73.05 ± 14.46^#^	0.103
*p* ^b^ (Baseline vs. Week 8)	0.011	0.005	
Week 12	67.98 ± 18.39*^#^	74.38 ± 14.07	0.013
*p* ^b^ (Baseline vs. Week 12)	< 0.001	0.109	

^a^ One‐tailed test denotes the test for (mean of Test Product − mean of Placebo) is smaller than zero using independent *t*‐test, where * denote statistically significant with *p*‐value < 0.05 for one‐tailed test.

^b^ One‐tailed test denotes the test for (mean of other weeks − mean of Week 0) is smaller than zero using a paired *t*‐test, where ^#^ denote statistically significant with *p*‐value < 0.05 for one‐tailed test.

**TABLE 5:** Comparison of the mean of wrinkle grading score between the test product and placebo.

Timepoint	Wrinkle scale (Mean ± SD)		*p* ^a^
	Test product (*n* = 66)	Placebo (*n* = 69)	
Week 0	2.76 ± 0.88	2.68 ± 0.87	0.61
Week 4	2.70 ± 0.87	2.64 ± 0.87	0.66
*p* ^b^ (Baseline vs. Week 4)	0.16	0.06	
Week 8	2.70 ± 0.88	2.63 ± 0.83	0.65
*p* ^b^ (Baseline vs. Week 8)	0.10	0.09	
Week 12	2.67 ± 0.91^#^	2.62 ± 0.84^#^	0.74
*p* ^b^ (Baseline vs. Week 12)	0.03	0.03	

^a^ Independent *t*‐test.

^b^ Paired *t*‐test, where ^#^ denote statistically significant with *p*‐value < 0.05 vs. Week 0.

### 3.5 Participant Self‐Assessment and Satisfaction

Participants completed a 14‐items self‐assessment questionnaire at week 4, 8 and 12 using a 5‐point Likert scale ranging from strongly agree (5), agree (4), neutral (3), disagree (2), and strongly disagree (1). The questionnaire evaluated perceived skin benefits, product acceptability, and tolerability. Overall, both groups demonstrated a progressive increase in mean scores over time, reflecting generally positive user experiences. However, the magnitude and pattern of improvement differed between groups (Table 6). At week 4, mean satisfaction scores were comparable between the test product and placebo groups for most questionnaire items. Slightly higher ratings in the placebo group were observed for several skin‐related perceptions (Q1–Q6), as well as taste‐ and palatability‐related items (Q9–Q11). By week 8, the test product group showed significant improvements from baseline on multiple skin efficacy items, including skin hydration (Q2), skin firmness (Q3), elasticity (Q4), and overall skin condition (Q8) (*p* < 0.05). These improvements indicate that participants began perceiving noticeable skin benefits around week 8. The placebo group also showed moderate increases in mean scores, though without statistically significant changes in most efficacy‐related items. Sensory attributes such as taste and ease of consumption (Q9–Q11) remained similarly rated between groups. At week 12, the test product group demonstrated the highest and most consistent satisfaction scores, with statistically significant improvements compared with week 4 and/or week 8 across nearly all skin‐related items (Q1–Q8), as indicated by *p < 0.05 vs. Week 4, ^#^ *p* < 0.05 vs. Week 8. Participants reported perceived benefits in skin youthfulness, hydration, elasticity, smoothness, radiance, and wrinkle reduction. While the placebo group demonstrated modest score elevations in various items, the only statistically significant in only Q4. Scores related to taste, color, pleasantness of consumption, gastrointestinal comfort, and perceived safety (Q9–Q14) remained high (> 3.9 on average) in both groups throughout the study. No significant between‐group differences were observed, suggesting that both formulations were well tolerated and acceptable for daily use.

**TABLE 6:** Comparison of self‐assessment of satisfaction score of the test product and placebo.

Questions	Week 4		Week 8		Week 12	
	Test product (*n* = 66)	Placebo (*n* = 69)	Test product (*n* = 66)	Placebo (*n* = 69)	Test product (*n* = 66)	Placebo (*n* = 69)
Q1: Skin youthfulness	3.182 ± 0.802	3.420 ± 0.775	3.439 ± 0.659	3.493 ± 0.740	3.758 ± 0.725*^#^	3.609 ± 0.712
Q2: Skin hydration	3.379 ± 0.818	3.623 ± 0.806	3.803 ± 0.706*	3.841 ± 0.779	4.167 ± 0.736*^#^^a^	3.899 ± 0.770
Q3: Skin firmness	3.273 ± 0.814^a^	3.551 ± 0.758	3.606 ± 0.677*	3.696 ± 0.773	3.985 ± 0.794*^#^	3.797 ± 0.759
Q4: Skin elasticity	3.273 ± 0.869	3.551 ± 0.796	3.636 ± 0.715*	3.725 ± 0.784	3.985 ± 0.690*^#^	3.870 ± 0.784*
Q5: Skin wrinkle appearance	3.167 ± 0.870	3.435 ± 0.866	3.424 ± 0.681	3.406 ± 0.880	3.742 ± 0.810*	3.725 ± 0.784
Q6: Skin smoothness	3.364 ± 0.888	3.638 ± 0.857	3.515 ± 0.638	3.696 ± 0.912	3.939 ± 0.782*^#^	3.899 ± 0.807
Q7: Skin radiance	3.364 ± 1.002	3.493 ± 0.885	3.621 ± 0.651	3.710 ± 0.893	3.970 ± 0.803*^#^	3.783 ± 0.838
Q8: Overall skin condition	3.333 ± 0.966^a^	3.667 ± 0.816	3.742 ± 0.686*	3.826 ± 0.785	4.015 ± 0.774*	3.870 ± 0.705
Q9: Product taste	3.682 ± 1.112	3.812 ± 1.061	4.015 ± 0.813	3.826 ± 1.057	4.227 ± 0.908*	3.986 ± 0.931
Q10: Product color	3.773 ± 1.078	4.087 ± 0.853	3.879 ± 0.851	3.986 ± 0.866	4.273 ± 0.795*^#^	4.217 ± 0.820
Q11: Ease of product consumption	3.803 ± 1.126	4.058 ± 0.889	3.894 ± 0.879	3.942 ± 0.938	4.288 ± 0.855*^#^	4.130 ± 0.839
Q12: Gastrointestinal tolerability	4.015 ± 1.234	4.333 ± 0.965	4.091 ± 1.003	4.275 ± 0.922	4.379 ± 0.799	4.420 ± 0.914
Q13: Absence of adverse effects	3.939 ± 1.251	4.261 ± 1.024	4.152 ± 0.996	4.319 ± 0.962	4.379 ± 0.780*	4.449 ± 0.814
Q14: Overall product satisfaction	4.091 ± 1.019	4.261 ± 0.995	4.167 ± 0.887	4.319 ± 0.849	4.424 ± 0.786	4.420 ± 0.830

*Note:* Repeated measure ANOVA with post‐hoc Tukey test, *p < 0.05 vs. Week 4, ^#^ *p* < 0.05 vs. Week 8. Unpaired *t*‐test, ^a^ *p* < 0.05 vs. placebo at the same time point.

### 3.6 Safety and Tolerability

Compliance with product consumption was high throughout the study. All participants who completed the study achieved an adherence rate of no less than 80%, based on returned sachet counts and participant diary records. Furthermore, no participant met the predefined non‐compliance criterion of missing five or more consecutive doses. Based on interviews conducted with participants during each study visit and a thorough review of their daily diaries, no adverse events directly related to product consumption were reported in either the test product or placebo group. Moreover, the safety monitoring was assessed by measuring vital signs, body mass index (BMI), and biochemical analyzes of blood and urine samples, including liver function, kidney function, and haematology, comparing between the baseline and after 12 weeks of product administration. The results showed that there was no significant difference for all parameters from both the placebo and test product groups at baseline and they were within the normal reference ranges, as presented in Table 7. After 12 weeks of product administration, comparisons between baseline and post‐intervention values revealed statistically significant changes in some parameters in both the placebo and test product groups (*p* < 0.05). However, the safety assessments identified that 9 participants in the test product group and 7 participants in the placebo group exhibited elevated urinary albumin‐to‐creatinine ratio (ACR) values exceeding the upper limit of the normal range (30 mg/g). Additionally, 3 participants in the placebo group presented with elevations in liver enzyme levels, specifically aspartate aminotransferase (AST) and alanine aminotransferase (ALT) beyond the upper reference limits of 37 U/L and 40 U/L, respectively. One participant in the placebo group demonstrated an increase in AST only, while one participant in the test product group exhibited an elevation in ALT only. These laboratory abnormalities occurred in a minority of participants across both treatment groups. There was no clear or consistent pattern that pointed to a link between the test product and the adverse events. Accordingly, the observed increase in values was not deemed clinically significant. Taken together, these findings support the conclusion that the test product was well tolerated over the 12‐week study period, with no clinically meaningful adverse effects attributable to its consumption.

**TABLE 7:** Comparison of blood and urine biochemistry for safety monitoring.

Parameter	Visit	Test product (*n* = 70)	Placebo (*n* = 70)	*p* ^a^	Reference range
		Mean ± SD	Mean ± SD		
Biochemical parameters					
BUN (mg/dL)	Week 0	11.03 ± 3.98	11.29 ± 3.59	0.69	7–18
	Week 12	10.95 ± 2.86	11.77 ± 3.96	0.16	
	*p* ^b^	0.860	0.291		
Serum creatinine (mg/dL)	Week 0	0.89 ± 0.15	0.92 ± 0.19	0.27	0.4–1.10
	Week 12	0.85 ± 0.19^#^	0.87 ± 0.21^#^	0.463	
	*p* ^b^	0.01	0.001		
eGFR (mL/min/1.73 m^2^)	Week 0	84.86 ± 14.36	83.32 ± 13.90	0.52	≥ 60
	Week 12	90.42 ± 14.67^#^	89.14 ± 16.19^#^	0.63	
	*p* ^b^	0.001	< 0.001		
Urine creatinine (mg/dL)	Week 0	93.16 ± 71.28	114.42 ± 86.44	0.12	29–226
	Week 12	81.60 ± 77.08	81.72 ± 63.30^#^	0.99	
	*p* ^b^	0.18	0.001		
Urine microalbumin (mg/dL)	Week 0	8.33 ± 4.63	9.52 ± 6.81	0.23	0–20
	Week 12	8.82 ± 8.68	7.87 ± 5.69^#^	0.44	
	*p* ^b^	0.66	0.04		
Albumin‐to‐creatinine ratio (ACR) (mg/gcr)	Week 0	12.48 ± 7.98	11.90 ± 11.87	0.74	0–30
	Week 12	17.73 ± 14.12^#^	18.80 ± 36.84	0.82	
	*p* ^b^	0.003	0.11		
Total protein (g/dL)	Week 0	7.79 ± 0.61	7.76 ± 0.55	0.77	6.4–8.3
	Week 12	7.84 ± 0.72	7.73 ± 0.63	0.33	
	*p* ^b^	0.55	0.66		
Albumin (g/dL)	Week 0	4.66 ± 0.45	4.63 ± 0.39	0.66	3.5–5.5
	Week 12	4.61 ± 0.52	4.69 ± 0.43	0.34	
	*p* ^b^	0.34	0.18		
Globulin (g/dL)	Week 0	3.13 ± 0.37	3.14 ± 0.39	0.97	1.9–3.5
	Week 12	3.24 ± 0.36*	3.05 ± 0.45^#^	0.01	
	*p* ^b^	0.06	0.02		
Total bilirubin (mg/dL)	Week 0	0.80 ± 0.26	0.79 ± 0.24	0.89	0.0–2.0
	Week 12	0.75 ± 0.23*	0.66 ± 0.21^#^	0.02	
	*p* ^b^	0.08	< 0.001		
Direct bilirubin (mg/dL)	Week 0	0.40 ± 0.15	0.39 ± 0.15	0.91	0.1–0.3
	Week 12	0.36 ± 0.12^#^	0.37 ± 0.14	0.70	
	*p* ^b^	0.05	0.221		
AST (U/L)	Week 0	21.24 ± 6.09	20.87 ± 7.04	0.74	0–37
	Week 12	20.46 ± 5.79	23.19 ± 10.07^#^	0.05	
	*p* ^b^	0.197	0.01		
ALT (U/L)	Week 0	21.36 ± 9.65	22.83 ± 11.80	0.42	0–40
	Week 12	19.59 ± 9.92	23.00 ± 10.07	0.13	
	*p* ^b^	0.08	0.89		
ALP (U/L)	Week 0	68.63 ± 19.07	66.76 ± 16.82	0.54	34–115
	Week 12	72.83 ± 20.84^#^	66.77 ± 17.00	0.06	
	*p* ^b^	0.003	0.99		
Haematology parameters					
Hemoglobin (g/dL)	Week 0	13.37 ± 1.47	13.15 ± 1.47	0.38	12.5–18.0
	Week 12	13.35 ± 1.51	13.33 ± 1.48^#^	0.94	
	*p* ^b^	0.76	0.02		
Hct (%)	Week 0	38.47 ± 3.78	37.91 ± 3.58	0.38	37–52
	Week 12	38.31 ± 3.91	38.71 ± 4.02^#^	0.55	
	*p* ^b^	0.53	0.002		
MCV (fL)	Week 0	81.51 ± 8.96	78.91 ± 8.93	0.09	78–100
	Week 12	81.59 ± 9.15	80.11 ± 9.08^#^	0.34	
	*p* ^b^	0.69	< 0.001		
MCH (pg)	Week 0	28.59 ± 3.72	27.54 ± 3.66	0.10	27–31
	Week 12	28.66 ± 3.90	27.86 ± 3.66^#^	0.21	
	*p* ^b^	0.36	< 0.001		
MCHC (g/dL)	Week 0	34.94 ± 1.18	34.80 ± 1.07	0.45	33–36
	Week 12	34.94 ± 1.26	34.64 ± 1.14	0.14	
	*p* ^b^	1.00	0.15		
RDW (%)	Week 0	13.43 ± 1.88	13.76 ± 1.86	0.30	11.5–14.0
	Week 12	13.40 ± 2.01	13.73 ± 1.85	0.31	
	*p* ^b^	0.70	0.73		
RBC (×10^6^/cu.mm)	Week 0	4.57 ± 0.67	4.84 ± 0.59	0.40	4.2–6.0
	Week 12	4.72 ± 0.66	4.85 ± 0.64	0.27	
	*p* ^b^	0.29	0.85		
Platelets (×10^3^) (cell/cu.mm)	Week 0	276.87 ± 65.44	288.30 ± 70.87	0.32	140–400
	Week 12	266.04 ± 60.26^#^	288.20 ± 69.45	0.05	
	*p* ^b^	0.02	0.98		
WBC (×10^3^) (cell/cu.mm)	Week 0	6.11 ± 14.66	6.57 ± 14.81	0.07	5000–10 000
	Week 12	5.91 ± 14.90	6.36 ± 16.91	0.09	
	*p* ^b^	0.13	0.22		
Neutrophil (%)	Week 0	51.73 ± 7.84	51.11 ± 8.23	0.65	50–65
	Week 12	50.86 ± 8.33	50.57 ± 10.15	0.86	
	*p* ^b^	0.38	0.60		
Lymphocyte (%)	Week 0	40.14 ± 7.43	40.64 ± 8.35	0.71	25–40
	Week 12	41.14 ± 8.03	41.96 ± 9.71	0.72	
	*p* ^b^	0.16	0.19		
Monocyte (%)	Week 0	6.26 ± 1.67	6.29 ± 1.42	0.91	3–9
	Week 12	6.27 ± 1.67	6.06 ± 1.68	0.45	
	*p* ^b^	0.94	0.30		
Eosinophil (%)	Week 0	1.83 ± 1.38	1.87 ± 1.48	0.86	1–3
	Week 12	1.43 ± 0.89^#^	1.40 ± 0.82^#^	0.84	
	*p* ^b^	0.02	0.002		
Basophil (%)	Week 0	0.04 ± 0.20	0.09 ± 0.28	0.31	0–1
	Week 12	0.03 ± 0.17	0.06 ± 0.23	0.41	
	*p* ^b^	0.32	0.42		
Vital sign and BMI					
Systolic blood pressure: SBP (mmHg)	Week 0	121.81 + 14.85	122.21 + 14.87	0.87	≤ 120
	Week 12	115.81 + 12.02^#^	115.34 + 15.92^#^	0.84	
	*p* ^b^	< 0.001	< 0.001		
Diastolic blood pressure: DBP (mmHg)	Week 0	79.04 + 9.13	79.07 + 9.92	0.99	≤ 80
	Week 12	76.79 + 8.25^#^	75.94 + 8.56^#^	0.54	
	*p* ^b^	0.02	< 0.001		
Heart rate: HR (bpm)	Week 0	79.27 + 10.44	79.20 + 9.51	0.97	60–100
	Week 12	77.69 + 9.50^#^	76.29 + 11.04	0.42	
	*p* ^b^	0.03	0.09		
Body mass index: BMI (kg/m^2^)	Week 0	23.42 + 2.42	23.74 + 2.77	0.46	≤ 25
	Week 12	23.42 + 2.45	23.75 + 2.70	0.45	
	*p* ^b^	0.45	0.55		

^a^ Independent *t*‐test.

^b^ Paired *t*‐test, where ^#^ denote statistically significant with *p*‐value < 0.05 vs. Week 0, and where * denote statistically significant with *p*‐value < 0.05 vs. placebo.

## 4 Discussion

This randomized placebo‐controlled trial investigated the efficacy of a hydrolyzed collagen supplement in improving skin health in healthy adult volunteers. The findings provide evidence of specific benefits on the skin hydration, elasticity, and wrinkle reduction. After 12 weeks of daily supplementation with HYCOS, a commercially formulated hydrolyzed collagen product containing hydrolyzed collagen peptides, glutathione, and vitamin E, the test product group showed statistically significant improvements in objective skin elasticity parameters (R2, R5, R7) when compared to baseline and, in some measures, when compared to the placebo group. The test product group also experienced a greater reduction in crow's feet wrinkle depth as measured by PRIMOS^CR^ 3D skin roughness, particularly initial roughness (R3z) relative to placebo. These results support the study's hypothesis that oral supplements containing collagen, glutathione, and vitamin E can enhance dermal properties associated with aging. In contrast, skin hydration was found to increase in both groups to a similar extent, with no significant between‐group difference, suggesting a more generalized or placebo‐related effect for that outcome. Both the test product and placebo treatments were well tolerated. Vital signs and blood parameters, including complete blood count and liver and kidney function markers, remained within normal clinical ranges.

The enhancement of skin elasticity observed with collagen, glutathione, and vitamin E combination supplementation is consistent with prior studies. Collagen peptides are thought to exert their effects by accumulating small peptides (e.g., prolyl‐hydroxyproline) in the dermis and stimulating fibroblast activity and extracellular matrix production [10, 11]. The improvements observed in skin elasticity and wrinkle parameters align with findings from previous RCTs assessing oral hydrolyzed collagen peptides. Proksch et al. [17] reported that 8 weeks of 2.5 g or 5 g daily hydrolyzed collagen peptides significantly increased skin elasticity in women aged 35–55 years. Fish‐derived collagen peptides showed significant benefits on skin elasticity and wrinkle reduction after 12 weeks [15, 16]. In this trial, we similarly found that HYCOS collagen supplementation led to improved biomechanical metrics (e.g., gross elasticity (R2) increased ~3%–4% from baseline), whereas the placebo showed slight change. Notably, the test product group had significantly higher gross elasticity than placebo as early as 4 weeks into treatment, indicating a relatively rapid onset of effect on skin's elastic properties, which related to Inoue et al. [10], also documented significant increases in skin elasticity measured by cutometer (R2) after 4 and 8 weeks of collagen hydrolysate ingestion, lending support that the time frame and magnitude of elasticity improvements in this study are plausible and reproducible. By week 12, slight improvements in elasticity were also noted in the placebo group, potentially reflecting lifestyle factors or seasonal influences, which reduced the magnitude of the between‐group difference. Nevertheless, the overall pattern continued to favour the test product group, which exhibited earlier and more pronounced gains in elasticity.

The HYCOS collagen supplement demonstrated a clear anti‐wrinkle effect, as shown by significant reductions in PRIMOS measured wrinkle depth and roughness, particularly in initial roughness (R3z). By week 12, the test product group exhibited smoother microtopography in the crow's feet region compared with the placebo group, consistent with improvements reported in prior studies. A randomized trial in 114 women (45–65 years) found that a specific collagen peptide led to a 20% reduction in eye wrinkle volume after 8 weeks [7]. In this study, the reduction in initial roughness (R3z), maximum roughness (Rmax), and total roughness (Rt) were more modest (~4%–5%), which may reflect differences in measurement methodology or population characteristics, as our cohort was slightly younger and included both women and men. Despite this, the directionality of the effect was consistent, indicating that collagen intake along with glutathione, and vitamin E in combination contributed to quantifiable wrinkle smoothing. Although the crow's feet wrinkle roughness measurement by PRIMOS demonstrated significant reductions in wrinkle microtopography particularly in R3z, and to a lesser degree in Rmax and Rt the clinical wrinkle scores showed only subtle improvements that were comparable between the test product and placebo groups. This divergence between objective instrumental measures and subjective clinical grading has been reported in previous trials of oral collagen peptides and other nutricosmetic interventions [15, 16]. Instrumental systems such as PRIMOS assess skin surface topography at the micrometer scale, enabling the detection of subtle changes in wrinkle depth, peak‐to‐valley variation, and localized roughness that are imperceptible to the naked eye. These early microstructural modifications likely reflect initial phases of dermal remodeling, such as increased fibroblast activity, enhanced collagen fibrillogenesis, and improved extracellular matrix organization, which typically precede changes that are visibly apparent on the skin surface. In contrast, clinical wrinkle grading relies on visual assessment, which is constrained by human perceptual thresholds, inter‐rater variability, lighting conditions, and the degree of change required to shift an ordinal score. Observable improvements generally emerge only when structural changes reach a magnitude sufficient to modify overall skin texture or wrinkle depth, a process that may require a longer intervention period or participants with greater baseline wrinkle severity. Consequently, despite the significant reductions in PRIMOS‐derived parameters, particularly in R3z and, to a lesser extent, Rmax and Rt, these microtopographic improvements did not manifest as visibly distinguishable changes on photographic grading. Taken together, the findings suggest that PRIMOS is capable of capturing early, quantifiable improvements in wrinkle roughness that may precede clinically visible wrinkle reduction. The photographic grading scale, by comparison, detected only subtle and comparable changes in both groups, underscoring its lower sensitivity for early microlevel improvements. This pattern supports the interpretation that dermal remodeling induced by collagen supplementation occurs progressively and may require a longer timeframe before becoming perceptible through visual clinical assessment. Although the magnitude of change in PRIMOS derived parameters was modest, these findings may still be considered clinically relevant because they reflect measurable improvements in skin surface microtopography. In populations with relatively mild baseline wrinkle severity, objective instrumental changes may represent early treatment responses that precede visually detectable wrinkle reduction.

Skin hydration improved substantially in both treatment arms, with no significant between‐group difference. Several previous trials of hydrolyzed collagen have reported superior hydration outcomes in collagen‐treated participants. Previously, RCT demonstrated that 8 weeks of collagen peptides ingestion significantly improved skin hydration and reduced trans‐epidermal water loss compared with placebo [10]. In the present study, although significant improvements in skin moisture were observed from baseline, the placebo beverage generated a comparable effect. Several factors may explain this outcome. The placebo contained citric acid and flavouring agents and required preparation with water, resulting in the daily consumption of approximately 150–200 mL of fluid. Increased water intake has been shown to improve stratum corneum hydration, particularly in individuals with low baseline moisture levels [25]. Additionally, this effect may also be attributable to ingredients common to both products, such as anhydrous citric acid, which has been reported to increase viable epidermal thickness and glycosaminoglycan content [26, 27], thereby reducing trans‐epidermal water loss and potentially enhancing skin hydration. Moreover, the observed increase in hydration may partly reflect behavioural changes during the study, as participants consistently consumed a daily beverage as part of the intervention. Such routine water intake and increased attention to skincare behaviours could have further contributed to improvements in skin hydration in both groups. Interestingly, although hydration did not differ between groups, this HYCOS formulation demonstrated clear advantages in dermal‐level outcomes, including elasticity and wrinkle roughness. These findings align with the established mechanisms of collagen peptides, which predominantly influence fibroblast activity, extracellular matrix synthesis, and dermal remodeling rather than stratum corneum hydration. Consequently, the absence of a between‐group difference in hydration supports the interpretation that HYCOS exerts its benefits primarily through structural improvements within the dermis rather than through superficial moisture enhancement. The notable placebo‐associated rise in hydration also underscores the importance of an active‐matched control in nutritional intervention trials.

No adverse effects were attributed to the HYCOS collagen formulation, supporting its safety for routine oral consumption. Hydrolyzed collagen peptides, particularly those used in collagen peptides‐based products, have been widely recognized as safe and well tolerated in human studies, with previous trials reporting no clinically relevant adverse events at doses of 2.5–10 g/day [16, 17, 28]. The present study further demonstrates that a daily dose of 6.5 g of HYCOS comprising collagen peptides together with glutathione and vitamin E was well tolerated over 12 weeks, with haematological and biochemical safety parameters remaining within normal clinical ranges. Although a small number of participants in both groups exhibited laboratory values slightly above upper reference limits (e.g., ACR, AST, ALT), these findings occurred at similar frequencies in both the test product and placebo groups and could not be attributed to or associated with HYCOS supplementation.

For the self‐assessment questionnaire, both groups reported progressively increasing satisfaction scores over the 12‐week period, reflecting positive user experience. The test product group showed clearer improvements in perceived skin benefits. Early ratings at week 4 were similar between groups, likely reflecting initial placebo effects, but by week 8 the test product group reported significant gains in hydration, firmness, elasticity, and overall skin condition, aligning with emerging biophysical improvements. This temporal pattern suggests that subjective perceptions of skin improvement developed alongside measurable changes in skin elasticity, supporting the clinical relevance of instrumental findings. By week 12, satisfaction scores in the test product group were highest and most consistent across skin‐related items, whereas the placebo group showed only modest increases, with statistical significance observed for elasticity alone. Sensory attributes, gastrointestinal comfort, and perceived safety remained favourable and comparable in both groups, indicating good tolerability and acceptability of the HYCOS beverage. Overall, the self‐assessment results complement objective measurements, suggesting that participants perceived meaningful improvements in skin quality with collagen, glutathione, and vitamin E combination supplementation. The consistency between subjective perceptions and objective elasticity outcomes strengthens the overall interpretation of treatment benefit.

Strengths of this study include its randomized, double‐blind, placebo‐controlled design; the use of standardized and well‐characterized collagen peptides; and the application of objective, instrument‐based measurements of skin elasticity and wrinkle roughness. The trial also incorporated comprehensive clinical and laboratory safety assessments and achieved high participant retention, enhancing the reliability of the findings. In addition, the inclusion of both dermatologist grading and participant self‐assessment offered complementary perspectives on perceived skin changes, providing a more complete evaluation of treatment effects. These findings have practical relevance for dermatologists, cosmetic scientists, and consumers seeking evidence‐based nutricosmetic options for early skin aging. Oral HYCOS hydrolyzed collagen may function as a non‐invasive adjunct to topical therapies and photoprotection. As interest in ingestible skincare continues to expand, clinically validated formulations such as HYCOS may play an increasingly vital role within multi‐modal anti‐aging regimens. Future research should evaluate longer‐term supplementation, investigate different dosing strategies, and incorporate mechanistic biomarkers of extracellular matrix remodeling. Comparative studies examining collagen source, molecular weight distribution, and multi‐ingredient formulations would help clarify structure–function relationships. Research in more diverse populations and across a wider range of photoaging severity is also needed to extend generalizability of the significance of active treatment.

## 5 Conclusion

This 12‐week randomized, double‐blind, placebo‐controlled trial demonstrated that daily HYCOS collagen supplementation is safe and effectively improves skin elasticity and wrinkle roughness, while hydration increased similarly across groups. These findings support the potential of this hydrolyzed collagen formulation, combined with glutathione and vitamin E, as a complementary approach for managing early signs of facial skin aging. Although not a substitute for established skincare and photoprotection practices, HYCOS may serve as a useful adjunct for individuals seeking non‐invasive strategies to enhance skin quality. Several limitations should be acknowledged. The study population comprised only Thai adults, which may limit generalizability to other ethnicities or individuals with more advanced photoaging. The 12‐week duration, although consistent with previous collagen trials, may not capture longer‐term or cumulative effects. Evaluation of a single dose of the HYCOS formulation also precluded assessment of dose–response relationships. Additionally, despite rigorous randomization and blinding, environmental or behavioural factors that may influence skin hydration cannot be fully ruled out.

## Author Contributions

**Sutthinee Wisutthathum:** writing – original draft, project administration, methodology, investigation, visualization, conceptualization. **Preeyanuch Pimjuk:** project administration, methodology, investigation, data curation. **Nitina Yeesibsean:** methodology, validation, conceptualization. **Katechan Jampachaisri:** formal analysis, conceptualization. **Panlop Chakkavittumrong:** methodology, wrinkle grading, conceptualization. **Warisara Pasri:** investigation. **Pichet Kittikun:** investigation. **Supaporn Tuanthai:** investigation. **Thanwarat Suebwongfag:** investigation. **Tanyarat Sutti:** investigation. **Jew Puay Tay:** resources, conceptualization. **Anjana Fuangchan:** methodology, formal analysis, investigation, data curation, supervision, conceptualization. **Neti Waranuch:** review and editing, methodology, visualization, funding acquisition, resources, supervision, conceptualization.

## Funding

This work was funded by Blackmores Institute, Australia. The sponsor provided the investigational products (HYCOS collagen and placebo) and financial support for the conduct of the trial. The sponsor had no role in the data analysis, the interpretation presented in the manuscript, or the decision to submit the manuscript for publication. This work was also partially supported by the Global and Frontier Research University Fund, Naresuan University (Grant No. R2566C053), Frontier Research and Innovation Cluster Fund, Naresuan University (Grant No. R2569C006), and the Centre of Excellence for Innovation in Chemistry (PERCH‐CIC), Commission on Higher Education, Ministry of Education is gratefully acknowledged.

## Ethics Statement

The protocol was approved by the Naresuan University Institutional Review Board (IRB) on 30 June 2022 (Certificate of Approval No. 0246/2022; IRB No. P1‐0070/2565), and the trial was conducted between July 2022 to July 2025. The trial was registered in the Thai Clinical Trials Registry: TCTR20250619006, (https://www.thaiclinicaltrials.org/show/TCTR20250619006), a member of the WHO Primary Network of ICTRP.

## Consent

All participants were provided with written informed consent prior to any study procedures.

## Conflicts of Interest

The authors declare no conflicts of interest.

## Data Availability

The data that support the findings of this study are not publicly available due to privacy and ethical considerations but may be obtained from the corresponding author upon reasonable request.

## References

[1] J. Krutmann , A. Bouloc , G. Sore , B. A. Bernard , and T. Passeron , “The Skin Aging Exposome,” Journal of Dermatological Science 85, no. 3 (2017): 152–161. 27720464 10.1016/j.jdermsci.2016.09.015

[2] M. A. Farage , K. W. Miller , P. Elsner , and H. I. Maibach , “Intrinsic and Extrinsic Factors in Skin Ageing: A Review,” International Journal of Cosmetic Science 30, no. 2 (2008): 87–95. 18377617 10.1111/j.1468-2494.2007.00415.x

[3] B. A. Gilchrest and J. Krutmann , Skin Aging (Springer Science & Business Media, 2006).

[4] J. Varani , M. K. Dame , L. Rittie , et al., “Decreased Collagen Production in Chronologically Aged Skin: Roles of Age‐Dependent Alteration in Fibroblast Function and Defective Mechanical Stimulation,” American Journal of Pathology 168, no. 6 (2006): 1861–1868. 16723701 10.2353/ajpath.2006.051302 PMC1606623

[5] G. J. Fisher , T. Quan , T. Purohit , et al., “Collagen Fragmentation Promotes Oxidative Stress and Elevates Matrix Metalloproteinase‐1 in Fibroblasts in Aged Human Skin,” American Journal of Pathology 174, no. 1 (2009): 101–114. 19116368 10.2353/ajpath.2009.080599 PMC2631323

[6] H. Al‐Atif , “Collagen Supplements for Aging and Wrinkles: A Paradigm Shift in the Fields of Dermatology and Cosmetics,” Dermatology Practical & Conceptual 12, no. 1 (2022): e2022018. 35223163 10.5826/dpc.1201a18 PMC8824545

[7] E. Proksch , M. Schunck , V. Zague , D. Segger , J. Degwert , and S. Oesser , “Oral Intake of Specific Bioactive Collagen Peptides Reduces Skin Wrinkles and Increases Dermal Matrix Synthesis,” Skin Pharmacology and Physiology 27, no. 3 (2013): 113–119. 24401291 10.1159/000355523

[8] K. Iwai , T. Hasegawa , Y. Taguchi , et al., “Identification of Food‐Derived Collagen Peptides in Human Blood After Oral Ingestion of Gelatin Hydrolysates,” Journal of Agricultural and Food Chemistry 53, no. 16 (2005): 6531–6536. 16076145 10.1021/jf050206p

[9] Y. Shigemura , D. Kubomura , Y. Sato , and K. Sato , “Dose‐Dependent Changes in the Levels of Free and Peptide Forms of Hydroxyproline in Human Plasma After Collagen Hydrolysate Ingestion,” Food Chemistry 159 (2014): 328–332. 24767063 10.1016/j.foodchem.2014.02.091

[10] N. Inoue , F. Sugihara , and X. Wang , “Ingestion of Bioactive Collagen Hydrolysates Enhance Facial Skin Moisture and Elasticity and Reduce Facial Ageing Signs in a Randomised Double‐Blind Placebo‐Controlled Clinical Study,” Journal of the Science of Food and Agriculture 96, no. 12 (2016): 4077–4081. 26840887 10.1002/jsfa.7606

[11] H. Ohara , S. Ichikawa , H. Matsumoto , et al., “Collagen‐Derived Dipeptide, Proline‐Hydroxyproline, Stimulates Cell Proliferation and Hyaluronic Acid Synthesis in Cultured Human Dermal Fibroblasts,” Journal of Dermatology 37, no. 4 (2010): 330–338. 20507402 10.1111/j.1346-8138.2010.00827.x

[12] M. Tanaka , Y. Koyama , and Y. Nomura , “Effects of Collagen Peptide Ingestion on UV‐B‐Induced Skin Damage,” Bioscience, Biotechnology, and Biochemistry 73, no. 4 (2009): 930–932. 19352014 10.1271/bbb.80649

[13] S. Y. Choi , E. J. Ko , Y. H. Lee , et al., “Effects of Collagen Tripeptide Supplement on Skin Properties: A Prospective, Randomized, Controlled Study,” Journal of Cosmetic and Laser Therapy 16, no. 3 (2014): 132–137. 24131075 10.3109/14764172.2013.854119

[14] S. Vleminckx , N. Virgilio , J. Asserin , J. Prawitt , and C. I. F. Silva , “Influence of Collagen Peptide Supplementation on Visible Signs of Skin and Nail Health and ‐Aging in an East Asian Population: A Double Blind, Randomized, Placebo‐Controlled Trial,” Journal of Cosmetic Dermatology 23, no. 11 (2024): 3645–3653. 39143887 10.1111/jocd.16458

[15] M. Lee , E. Kim , H. Ahn , S. Son , and H. Lee , “Oral Intake of Collagen Peptide NS Improves Hydration, Elasticity, Desquamation, and Wrinkling in Human Skin: A Randomized, Double‐Blinded, Placebo‐Controlled Study,” Food & Function 14, no. 7 (2023): 3196–3207. 36916504 10.1039/d2fo02958h

[16] M. Evans , E. D. Lewis , N. Zakaria , T. Pelipyagina , and N. Guthrie , “A Randomized, Triple‐Blind, Placebo‐Controlled, Parallel Study to Evaluate the Efficacy of a Freshwater Marine Collagen on Skin Wrinkles and Elasticity,” Journal of Cosmetic Dermatology 20, no. 3 (2021): 825–834. 32799362 10.1111/jocd.13676 PMC8176521

[17] E. Proksch , D. Segger , J. Degwert , M. Schunck , V. Zague , and S. Oesser , “Oral Supplementation of Specific Collagen Peptides Has Beneficial Effects on Human Skin Physiology: A Double‐Blind, Placebo‐Controlled Study,” Skin Pharmacology and Physiology 27, no. 1 (2014): 47–55. 23949208 10.1159/000351376

[18] R. B. de Miranda , P. Weimer , and R. C. Rossi , “Effects of Hydrolyzed Collagen Supplementation on Skin Aging: A Systematic Review and Meta‐Analysis,” International Journal of Dermatology 60, no. 12 (2021): 1449–1461. 33742704 10.1111/ijd.15518

[19] S. K. Myung and Y. Park , “Effects of Collagen Supplements on Skin Aging: A Systematic Review and Meta‐Analysis of Randomized Controlled Trials,” American Journal of Medicine 138, no. 9 (2025): 1264–1277. 40324552 10.1016/j.amjmed.2025.04.034

[20] G. Danessa , D. Notario , and R. Regina , “Effects of Collagen‐Based Supplements on Skin's Hydration and Elasticity: A Systematic Review and Meta‐Analysis,” Indian Journal of Dermatology, Venereology and Leprology 91 (2025): 730–740. 40826844 10.25259/IJDVL_1165_2023

[21] S. Y. Pu , Y. L. Huang , C. M. Pu , et al., “Effects of Oral Collagen for Skin Anti‐Aging: A Systematic Review and Meta‐Analysis,” Nutrients 15, no. 9 (2023): 2080. 37432180 10.3390/nu15092080 PMC10180699

[22] F. Watanabe , E. Hashizume , G. P. Chan , and A. Kamimura , “Skin‐Whitening and Skin‐Condition‐Improving Effects of Topical Oxidized Glutathione: A Double‐Blind and Placebo‐Controlled Clinical Trial in Healthy Women,” Clinical, Cosmetic and Investigational Dermatology 7 (2014): 267–274. 25378941 10.2147/CCID.S68424 PMC4207440

[23] T. F. Alzahrani , S. M. Alotaibi , A. A. Alzahrani , et al., “Exploring the Safety and Efficacy of Glutathione Supplementation for Skin Lightening: A Narrative Review,” Cureus 17, no. 1 (2025): e78045. 40013212 10.7759/cureus.78045 PMC11862975

[24] J. J. Thiele and S. Ekanayake‐Mudiyanselage , “Vitamin E in Human Skin: Organ‐Specific Physiology and Considerations for Its Use in Dermatology,” Molecular Aspects of Medicine 28, no. 5 (2007): 646–667. 17719081 10.1016/j.mam.2007.06.001

[25] L. Palma , L. T. Marques , J. Bujan , et al., “Dietary Water Affects Human Skin Hydration and Biomechanics,” Clinical, Cosmetic and Investigational Dermatology 8 (2015): 413–421. 26345226 10.2147/CCID.S86822 PMC4529263

[26] E. F. Bernstein , C. B. Underhill , J. Lakkakorpi , et al., “Citric Acid Increases Viable Epidermal Thickness and Glycosaminoglycan Content of Sun‐Damaged Skin,” Dermatologic Surgery 23, no. 8 (1997): 689–694. 9256916 10.1111/j.1524-4725.1997.tb00391.x

[27] C. M. Ditre , T. D. Griffin , G. F. Murphy , et al., “Effects of Alpha‐Hydroxy Acids on Photoaged Skin: A Pilot Clinical, Histologic, and Ultrastructural Study,” Journal of the American Academy of Dermatology 34, no. 2 Pt 1 (1996): 187–195. 8642081 10.1016/s0190-9622(96)80110-1

[28] D. Hexsel , V. Zague , M. Schunck , C. Siega , F. O. Camozzato , and S. Oesser , “Oral Supplementation With Specific Bioactive Collagen Peptides Improves Nail Growth and Reduces Symptoms of Brittle Nails,” Journal of Cosmetic Dermatology 16, no. 4 (2017): 520–526. 28786550 10.1111/jocd.12393

